# The Prognostic Value of Indoleamine-2,3-Dioxygenase Gene Expression in Urine of Prostate Cancer Patients Undergoing Radical Prostatectomy as First Treatment of Choice

**DOI:** 10.3389/fimmu.2020.01244

**Published:** 2020-08-14

**Authors:** Michael Thüring, Robin Knuchel, Ludovica Picchetta, Daniel Keller, Tobias S. Schmidli, Maurizio Provenzano

**Affiliations:** ^1^Oncology Research Unit, Department of Urology, University Hospital of Zurich, Zurich, Switzerland; ^2^Department of Immunology, University Hospital of Zürich, Zurich, Switzerland

**Keywords:** prostate cancer, IDO—indoleamine 2,3-dioxygenase, prognostic marker, liquid biopsy, radical prostatectomy, immune regulation, inflammation

## Abstract

Prostate cancer (PCa) is a slow-growing tumor representing one of the major causes of all new cancer cases and cancer mortality in men worldwide. Although screening methods for PCa have substantially improved, the outcome for patients with advanced PCa remains poor. The elucidation of the molecular mechanism that drives the progression from a slow-growing, organ-confined tumor to a highly invasive and castration-resistant PCa (CRPC) is therefore important. We have already proved the diagnostic potential of indoleamine-2,3-dioxygenase (IDO) when detected in urine of individuals at risk of developing PCa. The aim of this study was to implement IDO as a prognostic marker for PCa patients undergoing surgical treatment. We have thus conducted an observational study by collecting 100 urine samples from patients undergoing radical prostatectomy as first treatment of choice. To test the integrity of our investigation, scale dilution cells of an established PC3 cell line were added to urine of healthy donors and used for gene expression analysis by a TaqMan assay on the catalytic part of IDO mRNA. Our data show that the quantification of IDO mRNA in urine of patients has a very promising ability to identify patients at high risk of cancer advancement, as defined by Gleason score. Our goal is to lay the groundwork to develop a superior test for PCa. The data generated are thus necessary (i) to strengthen the IDO-based diagnostic/prognostic test and (ii) to provide patients and clinicians with an affordable and easy screening test.

## Introduction

Prostate cancer (PCa) represents the first leading cause of cancer morbidity and the third of cancer death in men in developed countries, with a worldwide incidence rate accounting for 14% of total newly diagnosed cases and a worldwide total cancer mortality rate of 6% (1). It is of relevance that prostate tissues appear to be characterized by features consistent with an immunosuppressive microenvironment. Infiltrating CD4^+^ and CD8^+^ T lymphocytes (TILs) are predominantly characterized by regulatory (2, 3) and functionally exhausted (PD-1+, B7-H1+) phenotypes (4–6). Furthermore, enhanced suppressive function of adaptive CD4^+^ Treg has been observed in the peripheral blood of patients with PCa and found to correlate with metastatic behavior (7).

The tumor microenvironment is the battlefield where not well-defined relationships between oncogenesis and immune surveillance to cancer take place. Tumor immune escape occurs through the secretion of different tumor-derived factors (TDFs) with immunosuppressive properties, such as indoleamine-2,3-dioxygenase (IDO). The specific IDO gene product plays a key role in tryptophan metabolism, and its enhanced activities might result in both the depletion of an amino acid essential for lymphocyte metabolism and in the generation of toxic metabolites (8).

The significant correlation between levels of expression of IDO and its activity (kynurenine/tryptophan ratio) in PCa specimens, the trend seen in relation to PCa patients' clinical features [Gleason score (GS)], and the finding that TGF-β expression was significantly correlated to IDO gene expression in PCa contributed to the identification of a peculiar subset of tumors (9). A high level of IDO has been reported to be correlated with poor clinical prognosis in cancers, such as ovarian cancer (10), endometrial cancer (11), colon carcinomas (12), malignant melanoma (13), and lung cancer (14). In renal cell carcinoma, levels of IDO mRNA in primary tumors or metastasis do not correlate with longer overall survival. In this specific setting, IDO was nearly exclusively expressed in endothelial cells of predominantly newly formed blood vessels, not in tumor cells, a condition that presumably inhibits tumor growth due to amino acid tryptophan depletion to cancer cells (15).

Currently, the selection of patients at risk of PCa and the indication for biopsy are based on the combination of prostate-specific antigen (PSA) blood test and the digital rectal examination (DRE) of the organ (16). Although PSA is the most commonly used screening parameter, it has a relatively poor specificity for PCa, which means that patients with benign prostate hyperplasia (BPH) or prostatitis might show higher PSA values and might therefore undergo unnecessary biopsies (17, 18).

To secure the diagnosis of PCa and to determine further therapeutic interventions, a fine-needle biopsy is necessary, although prostate sampling might be inconsistent at early stages (19, 20). In addition, complications like hematuria, hematospermia, and infection can occur during the procedure (21). Therefore, unnecessary costs and complications should be avoided for patients bearing no cancer lesions or an indolent form. A reliable biomarker that could identify without the use of biopsy patients with early aggressive or clinically significant tumors and rule them out while being easy to collect in a non-invasive procedure would be ideal (21, 22).

We have identified that the enzyme IDO could serve as a novel diagnostic biomarker for PCa in urine. We have developed a novel diagnostic approach based on the IDO mRNA and/or protein levels. Our current data show that the quantification of IDO mRNA in urine of patients has a very promising ability to identify patients harboring PCa (23). Because patients with higher expression of IDO in PCa at first diagnosis showed a significantly higher risk of tumor recurrence after prostatectomy, IDO may furthermore be used also as a recurrence marker.

Therefore, in this study, we evaluated the prognostic value of IDO gene expression in urine of PCa patients undergoing radical prostatectomy (RP) as first-line treatment. Urine was collected preoperatively, and results were correlated with clinicopathological characteristics.

## Materials and Methods

### Patients' Accrual and Clinicopathological Characteristics

We evaluated a case series of 100 patients bearing PCa at first diagnosis and undergoing RP as first treatment of choice in our institution between June 2016 and June 2017. Relevant clinical data were collected by reviewing patients' files. Clinicopathological parameters (PSA levels, tumor stage, and GS were assigned according to European Association of Urology (EAU) guidelines for PCa; uroweb.org/guidelines/prostate-cancer/). Local ethics committee approval and written informed consent from patients were obtained in accordance with the requirements of the Ethical Committee of Zürich (BASEC_2018-02101).

### Urine Processing

Urine of 20 to 50 ml was voided in DNA/RNA preservative cups (Sierra Diagnostic, USA) before RP. Depending on amount of urine collected, two-way processing was carried out: (i) for 20–50 cm^3^ of urine, the extraction of RNA was performed by using urine pellet generated after urine centrifugation at 2,000 rpm for 10 min at 4°C. (ii) For limited amount of urine (<20 cm^3^), the quantification of cell-free RNA was considered, and urine samples were aliquoted at 500 μl each test. Either pellet or cell-free RNA urine was treated with 700 μl of lysis solution (Ambion, USA), stored at −80°C or immediately used for RNA extraction (Ambion, USA). Total urine was used to test IDO enzymatic activity through the l-kynurenine/tryptophan ratio, as analyzed by ELISA (Immundiagnostik).

### Established Prostate Cancer Cell Line Spiked in Patients' Urine

PC3 is an established cells line from bone metastasis and produces a high level of IDO constitutively (23). PC3 was cultured in Roswell Park Memorial Institute (RPMI) 1640 medium containing 2 mM of l-glutamine (Invitrogen, Carlsbad, CA) together with 10% fetal bovine serum (Atlanta Biologicals, Lawrenceville, GA), 100 U/ml of penicillin, and 100 μg/ml of streptomycin. Urine from healthy donors spiked with PC3 was used as control or to validate our system. Accordingly, IDO gene expression was tested by plating PC3 cell line in four different sized growing areas (150, 75, 25, and 6 cm^2^) and cultured for 72 h, as previously described by us in Poyet et al. (24). Cells were harvested at about 90% confluence. Scale dilution of cells was added to urine of HD, and pellet was used for gene expression.

### Gene Expression Analysis

Total RNA extraction was performed by using the RNAqueous Kit according to the manufacturer's protocol (Applied Biosystems, USA). After extraction, RNA undertook DNase treatment and was subsequently retrotranscribed into cDNA (High Capacity cDNA Reverse Transcription Kit, Applied Biosystems). Quantitative gene amplification (qRT-PCR) was set up according to standard real-time PCR protocols using a Corbett Life Science Rotor-gene 3000 instrument (Corbett Life Science, Sydney, Australia) using TaqMan® Universal PCR Master Mix Reagents Kit (Labgene) and “on demand” sets of primers and probes for housekeeping genes (RNA ribosomal 18S and β-actin) (Thermo Fisher, Switzerland). The IDO assay (Custom TaqMan primers and probe design; Applied Biosystems) was designed to cover the exon–exon junction between exons 9 and 10. Primers were designed to allow a melting temperature of between 58 and 61°C, with an optimal length of 20 bp and CG content between 30 and 80%. The probe was designed not to start with G and in order to have a melting temperature 10°C higher than the one of the primers. A 3′ minor grove binder-probe [non-fluorescent quencher fitting the 5(6)-carboxyfluorescein (FAM) spectral qualities] was used. Primers and probe were used at a final concentration of 400 and 200 nM, respectively. TaqMan assay sequences are described in patent “INDOLEAMINE-2,3-DIOXYGENASE ASSAY FOR PROSTATE CANCER DIAGNOSIS AND PROGNOSIS” https://worldwide.espacenet.com/publicationDetails/biblio?II=0&ND=3&adjacent=true&locale=en_EP&FT=D&date=20180419&CC=WO&NR=2018069494A1&KC=A1#.

One microliter of cDNA was loaded into the TaqMan reaction mix, and reactions were run in a final volume of 20 μl. The reaction conditions were set accordingly to the manufacturer's instructions (TaqMan Gene Expression Master Mix, Applied Biosystems, USA). The absolute quantification of each gene's copies was calculated through the generation of a standard curve by serial dilution of synthetic oligonucleotides. Data were then given in copies per milliliter of urine. Where appropriate, RNA ribosomal 18S and β-actin were used as endogenous reference genes, and normalized data were be analyzed by the 2^−ΔΔCt^ method.

### Measurement of Tryptophan, Kynurenine, and Quinolinic Acid Concentration

IDO activity was measured in urine and performed as l-kynurenine vs. l-tryptophan (Kyn/Trp ratio) or quinolinic acid A vs. l-tryptophan (Q-A/Trp ratio) concentrations by ELISA according to manufacturer's instructions (Immundiagnostik, Bensheim, Germany). In addition, IDO protein release in urine of patients was analyzed as well by ELISA (Immundiagnostik, Bensheim, Germany).

### Statistical Analysis

Statistical analysis was performed with GraphPad Prism (v5.1) and SPSS (v23). Non-parametric tests for gene expression levels (Mann–Whitney *U*-test and Kruskal–Wallis test) were run. Categorical variables were evaluated by contingency table analyses and Pearson chi-square test or Fisher exact test, as appropriate. Two-sided *p* < 0.05 (95% CI) were considered statistically significant. The performance of IDO as prognostic factor in PCa was evaluated by calculating the area under the receiver operating characteristic (ROC) curve (AUC). Previous cutoffs were confirmed by sensitivity, specificity, positive predictive value (PPV), and negative predictive value (NPV) of the test.

## Results

### Indoleamine-2,3-Dioxygenase Gene Expression in Urine Represents a Valuable Test for Prostate Cancer Prognosis by Liquid Biopsy

To test the integrity of our investigation, which is the positive correlation between IDO gene expression in urine and number of cancer cells, PC3 cells were spiked in urine of healthy donors at various concentrations, as described in the *Materials and Methods*. To standardize IDO gene expression, the number of harvested cells was correlated with the amount of total RNA extracted after cell harvesting.

**Figure 1:**
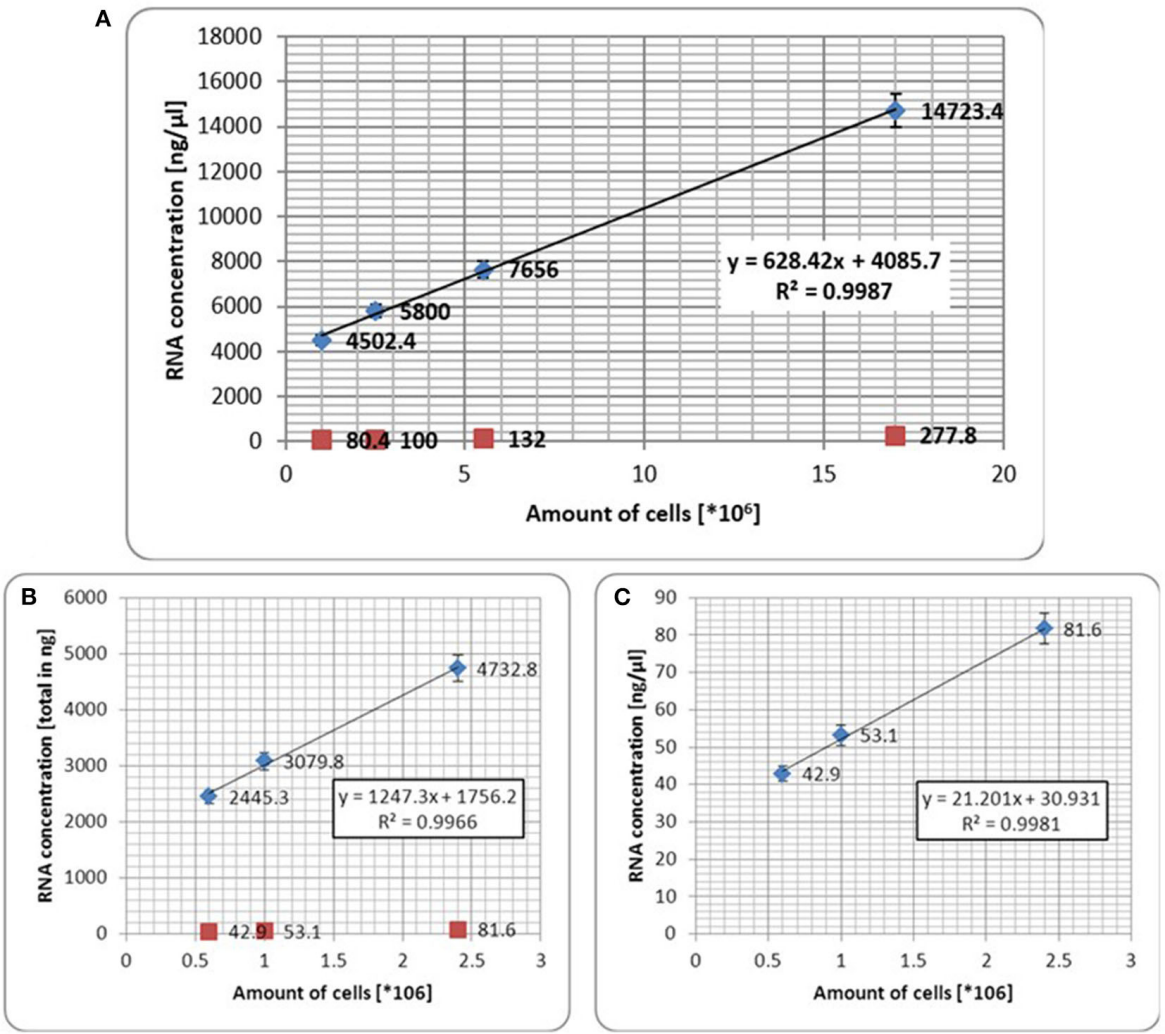
**(A)** Correlation between scale dilution of established PC3 cells line and total RNA extraction. Red dots represent number of seeded cells *per* growing area (6, 25, 75, and 150 cm^2^, respectively); blue dots represent amount of extracted total RNA from harvested cells *per* growing area (6, 25, 75, and 150 cm^2^, respectively). The test shows that correlation between number of harvested cells <1 × 10^6^ and total RNA **(B)** or RNA in ng/μl **(C)** was markedly confirmed. Error bars represent the mean ± SD of three replicates.

**Figure 2:**
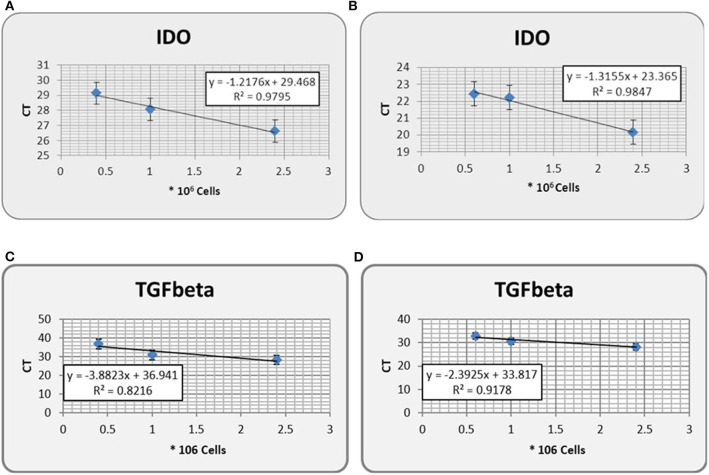
IDO gene expression (in CT, threshold cycle) from non spiked PC3 urine of healthy donors **(A)**, as compared with its expression from PC3-spiked counterpart **(B)**. Gene expression of TGF-β, a tumor-derived soluble factor involved in PCa progression, in either non spiked PC3 **(C)** or PC3-spiked **(D)** urine, has been used as control. Error bars represent the mean ± SD of three replicates. IDO, indoleamine-2,3-dioxygenase.

We first observed a positive correlation between number of cells harvested and total RNA extracted (Figure 1A). We additionally observed that the correlation between number of cells <1 × 10^6^ and RNA concentration, either total (Figure 1B) or in ng/μl (Figure 1C) was markedly confirmed (R^2^ < 0.9). To mimic prostate massage yield, the same numbers of cells were spiked into urine. We observed that IDO gene expression was confirmed as equal as IDO gene expression from non-spiked cells (Figures 2A,B), although ΔCt between spiked and non-spiked urine was of about 2logs (*p* > 0.5). In addition, IDO gene expression showed a linear regression value higher than TGF-β gene expression, a cytokine constitutively expressed in PC3 cell lines (Figures 2C,D). Despite spiked urine showing higher IDO gene expression, the latter is not markedly strong to justify the use of prostate massage for IDO testing.

### Detection of Indoleamine-2,3-Dioxygenase Gene in Urine Does Not Need Prostate Massage and Can Be Performed Either by Urine Pellet or by Cell-Free RNA

**Figure 3:**
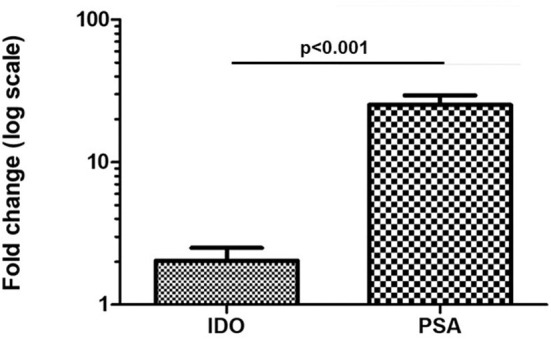
Fold change in IDO and PSA gene expression in five PCa patients before/after DRE. Amount of IDO mRNA is significantly maintained in urine of PCa patients undergoing DRE (~2-fold-increase), as compared with its expression in urine of same PCa patients before DRE. Differently, PSA value abundantly increased over 2-fold. Error bars represent the mean ± SD of three replicates. IDO, indoleamine-2,3-dioxygenase; PSA, prostate-specific antigen; PCa, prostate cancer; DRE, digital rectal examination.

One of the major limitations in performing gene expression in urine is the amount of RNA. For PCa diagnosis, this hurdle can be overcome by squeezing the organ through a prostate massage and extracting RNA from urine pellet. However, prostate massage is not always feasible. We analyzed IDO gene expression in urine of five patients undergoing PCa standard diagnosis (PSA level + DRE) collected before and after DRE. PSA gene expression was used as control. We observed that change in IDO gene expression was abundantly lower (~2-fold) than that of PSA (30-fold; *p* < 0.001; Figure 3). In addition, no significant differences for IDO gene expression were observed between pellet and cell-free RNA of urine collected before DRE (data not shown).

### The Prognostic Potential of Indoleamine-2,3-Dioxygenase for Diagnosed Prostate Cancer Can Redirect Treatment Options

**Figure 4:**
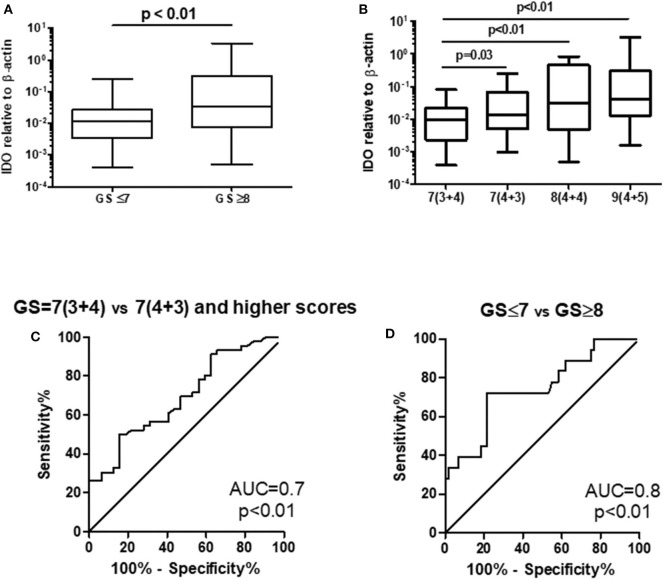
IDO gene expression correlated with Gleason score either **(A)** when comparing GS ≤ 7 vs. GS ≥ 8 or **(B)** when expressing single GS patterns. A ROC (AUC 0.7) **(C)** that compared Gleason score 7 (3 + 4) vs. 7 (4 + 3)/higher failed to confirm a previously defined cutoff for discriminating indolent vs. aggressive PCa, while a ROC (AUC 0.8) **(D)** that compared GS ≤ 7 vs. GS ≥ 8 did confirm a previous cutoff for discriminating indolent vs. aggressive PCa. IDO, indoleamine-2,3-dioxygenase; GS, Gleason score; ROC, receiver operating characteristic; AUC, area under the ROC curve; PCa, prostate cancer.

Out of 100 patients enrolled in this study, 20 were excluded owing to lack of complete clinicopathological parameters (TNM and GS after RP) and limited amount of material for IDO gene expression (rRNA 18S almost negligible; Ct > 30). By distributing patients on the basis of GS (GS ≤ 7; *n* = 60 and GS ≥ 8; *n* = 20), we found a significant variation for IDO gene expression in urine (GS ≤ 7 = mean 0.029 ± 0.046; median 0.012; GS ≥ 8 = 0.031 ± 0.075; median 0.033; *p* < 0.01; Figure 4A). To better determine the association between IDO gene expression in urine and cancer aggressiveness, we distributed patients on the basis of all GS patterns from GS = 7 to GS = 10 (no GS = 6 was reported, because indolent patients preferably undergo active surveillance). Therefore, we grouped our patients as follows: GS = 7 (3 + 4), *n* = 32; GS = 7 (4 + 3), *n* = 28; GS = 8, *n* = 12; and GS = 9 (4 + 5), *n* = 8. No PCa with GS = 9 (5 + 4) or GS = 10 was reported in our group of patients. A Kruskal–Wallis test was initially run to test mean variations among groups (*p* < 0.01). We found a significant difference of IDO gene expression in urine between patients with GS = 7 (3 + 4) and GS = 7 (4 + 3) (*p* = 0.03), GS = 8 (*p* < 0.01), and GS = 9 (4 + 5) (*p* < 0.01), whereas no significant differences were observed between GS = 7 (4 + 3) and higher scores [GS = 8 and GS = 9 (4 + 5)] (Figure 4B). Notably, no significant differences were observed for TNM distribution (data not shown).

To confirm the cutoff value previously defined (23), we run two ROC curves tests comparing PCa with GS = 7 (3 + 4) vs. GS = 7 (4 + 3)/higher score and GS ≤ 7 vs. GS ≥ 8. The AUC to dichotomize patients with indolent or aggressive PCa on the basis of on GSs either partially confirmed or ameliorated the sensitivity and specificity of the test calculated with the cutoff level defined previously (0.0096). Indeed, for GS = 7 (3 + 4) vs. GS = 7 (4 + 3)/higher score, the best cutoff level was 0.0123 (sensitivity 61% and specificity 60%), whereas at the cutoff level of 0.0096, the sensitivity was 70% and the specificity 53% (Figure 4C). Differently, for GS ≤ 7 vs. GS ≥ 8, the best cutoff level was 0.0288 (sensitivity 71% and specificity 78%), whereas at cutoff level of 0.0096, the sensitivity was 61% and the specificity was 60% (Figure 4D).

### Indoleamine-2,3-Dioxygenase Enzymatic Activity Might Predict Prostate Cancer Clinical Outcome

**Figure 5:**
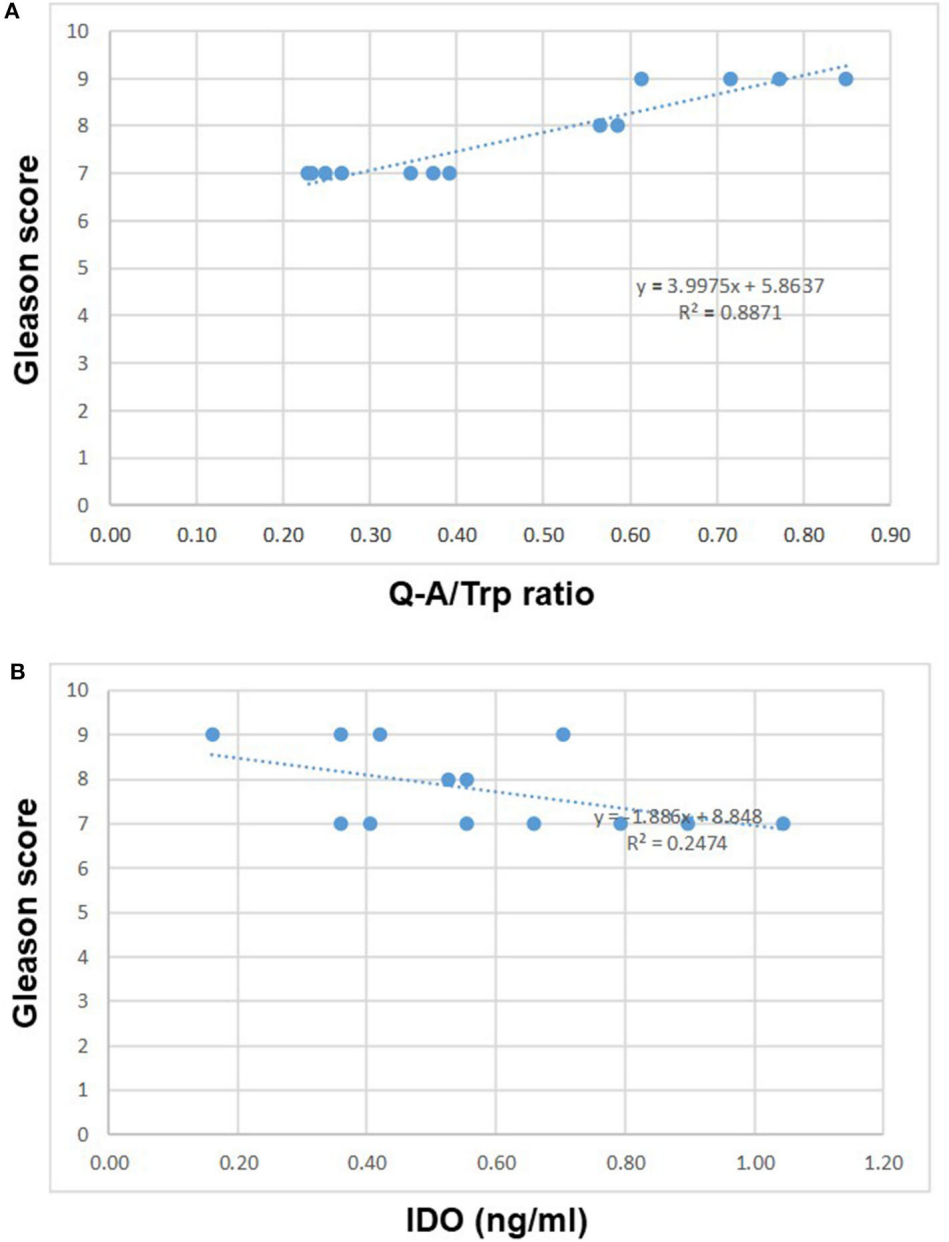
IDO activity characterized by Q-A/Trp ratio in 15 selected PCa with different GS (7–9) correlated with GS (*R*^2^ 0.88, *p* < 0.001 **(A)**. Differently, IDO protein release (ng/ml) inversely correlated with GS **(B)**. IDO, indoleamine-2,3-dioxygenase; PCa, prostate cancer; GS, Gleason score.

To confirm previous finding, the IDO activity (Kyn/Trp ratio or Q-A/Trp ratio) and IDO protein release were analyzed in 15 selected PCa patients with different GS (7 to 9). In keeping with IDO mRNA expression in urine, IDO activity characterized by Q-A/Trp ratio correlated with GS (7–9) (*R*^2^ 0.88, *p* < 0.001; Figure 5A), whereas activity by Kyn/Trp ratio did not (data not shown). In addition, IDO protein release (ng/ml) showed a tendency with GS (7–9) (*p* = 0.07), although the correlation was inversed and not significant (*R*^2^ 0.24, *p* = 0.3; Figure 5B).

## Discussion

PCa is the most frequently diagnosed cancer among men in first-world countries and one of the leading causes of cancer-related death in men (22).

The management of PCa is still difficult, as indolent tumors often do not have any effect on the patient's life expectancy and therefore need a minimum or no treatment. In contrast, aggressive forms can grow and metastasize quickly, which often leads to life-impairing consequences (25). For clinical stratification, the PSA, tumor size, and GS are being used.

Recently, urine-based biopsy has been considered the gold standard owing to the non-invasive procedure for their collection. Indeed, there are many new approaches that use urine as liquid biopsy for PCa diagnosis/prognosis, such as circulating tumor cells (26) and cell-free DNA (27) tests.

Recent studies showed the potential of IDO as such a novel marker (9, 23). The enzyme IDO is expressed in many tumor types including PCa and seems to contribute to the tumor's immunosuppressive abilities by converting tryptophan into kynurenine (28–30). It was shown that immunohistochemically measured IDO gene expression in prostate biopsies is highly specific for PCa (9). However, a clinically more viable way of measuring is required. A urine-based analysis of IDO RNA expression of men at risk of PCa development showed that it could reduce the number of unnecessary biopsies by 14.8–66.6% depending on the cutoff level (23).

Clinicians need a reliable, easy-to-handle, and affordable screening test. The IDO mRNA-based assay is possible in clinical/practitioner's routine. In our opinion, this IDO gene expression analysis in urine of PCa patients is stronger than a protein test in terms of sensitivity and affordability. Further investigation on larger cohort of patients is needed to confirm the results achieved at both studies with enough statistical power; to finalize the final procedures for a better detection of IDO; to rate with accurate estimation the sensitivity of this test; and to evaluate in more detail the significance of IDO localization in biopsies as a marker for the risk of biochemical recurrence.

The PSA blood test, despite being the gold standard for PCa active surveillance and recurrence, has several limitations for screening patients at risk of PCa. This is due to the following: (i) PSA is organ specific but not tumor specific. Elevated levels of PSA can be found in other pathologies (benign prostatic hyperplasia, and acute prostatitis) or in physiological conditions, such as age increase and sexual activity; (ii) PSA test lacks sensitivity because only 25% of patients with PSA of between 2.5 and 10 ng/ml, *gray zone*, show positive biopsies; and (iii) PSA is not indicative of tumor staging and grading alone.

Improving current methods for screening of patients is therefore of great importance and will positively affect a significant part of the population. We have identified IDO as an important player in the mechanisms that regulate PCa progression and clarified its role and activity in these settings. Tumor cells expressing IDO have a higher chance of escaping the immune surveillance, and due to the presence of IFNγ and TNFα in the tumor microenvironment, they have a greater ability to migrate and invade. Furthermore, patients with higher expression of IDO in PCa at first diagnosis showed a significantly higher risk of tumor recurrence after prostatectomy.

Particularly in PCa, the expression of tumor-derived soluble factors (TDSFs) able to impair the functions of immune system in patients has been defined by us in Banzola et al. (23). In this context, we previously observed that cytokine possibly involved in PCa progression (such as IL-6) was found significantly higher expressed in PCa specimens, as compared with BPH (9). This information was confirmed by cytokine detection in sera of PCa patients (31).

In addition, arginase production by macrophages infiltrating prostatic tissues has been shown to favor the induction of anergy in resident lymphocytes (4). In this context, cytokines might play a relevant role in coordinating cancer immunoediting (32).

In conclusion, we proved that the quantification of IDO mRNA in the urine of PCa patients is a potential prognostic tool for this malignancy. This dataset is large enough to support the use of IDO as a recurrent marker in PCa patients undergoing RP as the first treatment of choice. However, in order to strengthen IDO as a robust prognostic marker for the identification of patients at higher risk of PCa recurrence, additional data on clinical samples from a larger cohort and the improvement of data acquisition for a more reliable IDO enzymatic activity are needed. Thus, the IDO detection in the urine of PCa patients could contribute, together with the standard parameters for the diagnosis/prognosis of this disease, to improve its outcome.

## Data Availability Statement

The datasets generated for this study are available on request to the corresponding author.

## Ethics Statement

The studies involving human participants were reviewed and approved by Ethical Committee of Zürich (BASEC_2018-02101). The patients/participants provided their written informed consent to participate in this study.

## Author Contributions

MP conceived the study and supervised the project. MT, DK, RK, LP, and TS performed the experiments. MT, DK, RK, LP, and MP analyzed the data. MT, RK, and TS performed sample collection. MT, RK, TS, and MP arranged clinical aspect. TS and MP wrote informed consent. MT and MP wrote the paper. All authors contributed to the article and approved the submitted version.

## Conflict of Interest

The authors declare that the research was conducted in the absence of any commercial or financial relationships that could be construed as a potential conflict of interest.

## References

[1] Jemal A Bray F Center MM Ferlay J Ward E Forman D Global cancer statistics. CA Cancer J Clin. (2011) 61:69–90. 10.3322/caac.20107 21296855

[2] Kiniwa Y Miyahara Y Wang HY Peng W Peng G Wheeler TM. CD8+ Foxp3+ regulatory T cells mediate immunosuppression in prostate cancer. Clin Cancer Res. (2007) 13:6947–58. 10.1158/1078-0432.CCR-07-0842 18056169

[3] Miller AM Lundberg K Ozenci V Banham AH Hellstrom M Egevad L. CD4+CD25high T cells are enriched in the tumor and peripheral blood of prostate cancer patients. J Immunol. (2006) 177:7398–405. 10.4049/jimmunol.177.10.7398 17082659

[4] Bronte V Kasic T Gri G Gallana K Borsellino G Marigo I. Boosting antitumor responses of T lymphocytes infiltrating human prostate cancers. J Exp Med. (2005) 201:1257–68. 10.1084/jem.20042028 15824085 PMC2213151

[5] Ebelt K Babaryka G Frankenberger B Stief CG Eisenmenger W Kirchner T. Prostate cancer lesions are surrounded by FOXP3+, PD-1+ and B7-H1+ lymphocyte clusters. Eur J Cancer. (2009) 45:1664–72. 10.1016/j.ejca.2009.02.015 19318244

[6] Sfanos KS Bruno TC Meeker AK De Marzo AM Isaacs WB Drake CG. Human prostate-infiltrating CD8+ T lymphocytes are oligoclonal and PD-1+. Prostate. (2009) 69:1694–703. 10.1002/pros.21020 19670224 PMC2782577

[7] Yokokawa J Cereda V Remondo C Gulley JL Arlen PM Schlom J. Enhanced functionality of CD4+CD25(high)FoxP3+ regulatory T cells in the peripheral blood of patients with prostate cancer. Clin Cancer Res. (2008) 14:1032–40. 10.1158/1078-0432.CCR-07-2056 18281535

[8] Weber WP Feder-Mengus C Chiarugi A Rosenthal R Reschner A Schumacher R Differential effects of the tryptophan metabolite 3-hydroxyanthranilic acid on the proliferation of human CD8+ T cells induced by TCR triggering or homeostatic cytokines. Eur J Immunol. (2006) 36:296–304. 10.1002/eji.200535616 16385630

[9] Feder-Mengus C Wyler S Hudolin T Ruszat R Bubendorf L Chiarugi A. High expression of indoleamine 2,3-dioxygenase gene in prostate cancer. Eur J Cancer. (2008) 44:2266–75. 10.1016/j.ejca.2008.05.023 18619832

[10] Okamoto A Nikaido T Ochiai K Takakura S Saito M Aoki Y. Indoleamine 2,3-dioxygenase serves as a marker of poor prognosis in gene expression profiles of serous ovarian cancer cells. Clin Cancer Res. (2005) 11:6030–9. 10.1158/1078-0432.CCR-04-2671 16115948

[11] Ino K Yoshida N Kajiyama H Shibata K Yamamoto E Kidokoro K. Indoleamine 2,3-dioxygenase is a novel prognostic indicator for endometrial cancer. Br J Cancer. (2006) 95:1555–61. 10.1038/sj.bjc.6603477 17117179 PMC2360726

[12] Brandacher G Perathoner A Ladurner R Schneeberger S Obrist P Winkler C. Prognostic value of indoleamine 2,3-dioxygenase expression in colorectal cancer: effect on tumor-infiltrating T cells. Clin Cancer Res. (2006) 12:1144–51. 10.1158/1078-0432.CCR-05-1966 16489067

[13] Speeckaert R Vermaelen K van Geel N Autier P Lambert J Haspeslagh M. Indoleamine 2,3-dioxygenase, a new prognostic marker in sentinel lymph nodes of melanoma patients. Eur J Cancer. (2012) 48:2004–11. 10.1016/j.ejca.2011.09.007 22033321

[14] Astigiano S Morandi B Costa R Mastracci L D'Agostino A Ratto GB. Eosinophil granulocytes account for indoleamine 2,3-dioxygenase-mediated immune escape in human non-small cell lung cancer. Neoplasia. (2005) 7:390–6. 10.1593/neo.04658 15967116 PMC1501151

[15] Riesenberg R Weiler C Spring O Eder M Buchner A Popp T. Expression of indoleamine 2,3-dioxygenase in tumor endothelial cells correlates with long-term survival of patients with renal cell carcinoma. Clin Cancer Res. (2007) 13:6993–7002. 10.1158/1078-0432.CCR-07-0942 18056175

[16] Merriel SWD Funston G Hamilton W. Prostate cancer in primary care. Adv Ther. (2018) 35:1285–94. 10.1007/s12325-018-0766-1 30097885 PMC6133140

[17] Zambon CF Basso D Prayer-Galetti T Navaglia F Fasolo M Fogar P. Quantitative PSA mRNA determination in blood: a biochemical tool for scoring localized prostate cancer. Clin Biochem. (2006) 39:333–8. 10.1016/j.clinbiochem.2006.02.001 16516186

[18] Catalona WJ Southwick PC Slawin KM Partin AW Brawer MK Flanigan RC. Comparison of percent free PSA, PSA density, and age-specific PSA cutoffs for prostate cancer detection and staging. Urology. (2000) 56:255–60. 10.1016/S0090-4295(00)00637-3 10925089

[19] Lee DH Nam JK Park SW Lee SS Han JY Lee SD. Visually estimated MRI targeted prostate biopsy could improve the detection of significant prostate cancer in patients with a PSA level <10 ng/mL. Yonsei Med J. (2016) 57:565–71. 10.3349/ymj.2016.57.3.565 26996553 PMC4800343

[20] Klein EA Cooperberg MR Magi-Galluzzi C Simko JP Falzarano SM Maddala T A 17-gene assay to predict prostate cancer aggressiveness in the context of Gleason grade heterogeneity, tumor multifocality, biopsy undersampling. Eur Urol. (2014) 66:550–60. 10.1016/j.eururo.2014.08.001 24836057

[21] Loeb S Vellekoop A Ahmed HU Catto J Emberton M Nam R. Systematic review of complications of prostate biopsy. Eur Urol. (2013) 64:876–92. 10.1016/j.eururo.2013.05.049 23787356

[22] Fitzmaurice C Akinyemiju TF Al Lami FH Alam T Alizadeh-Navaei R Allen C. Global, regional, and national cancer incidence, mortality, years of life lost, years lived with disability, and disability-adjusted life-years for 29 cancer Groups, 1990 to 2016: a systematic analysis for the global burden of disease study. JAMA Oncol. (2018) 4:1553–68. 10.1200/JCO.2018.36.15_suppl.1568 29860482 PMC6248091

[23] Banzola I Mengus C Wyler S Hudolin T Manzella G Chiarugi A. Expression of indoleamine 2,3-Dioxygenase induced by IFN-gamma and TNF-alpha as potential biomarker of prostate cancer progression. Front Immunol. (2018) 9:1051. 10.3389/fimmu.2018.01051 29896191 PMC5986916

[24] Poyet C Thomas L Benoit TM Delmo DA Luberto L Banzola I Implication of vascular endothelial growth factor A and C in revealing diagnostic lymphangiogenic markers in node-positive bladder cancer. Oncotarget. (2017) 8:21871–883. 10.18632/oncotarget.15669 28423532 PMC5400630

[25] Lalonde E Ishkanian AS Sykes J Fraser M Ross-Adams H Erho N. Tumour genomic and microenvironmental heterogeneity for integrated prediction of 5-year biochemical recurrence of prostate cancer: a retrospective cohort study. Lancet Oncol. (2014) 15:1521–32. 10.1016/S1470-2045(14)71021-6 25456371

[26] Scher HI Heller G Molina A Attard G Danila DC Jia X. Circulating tumor cell biomarker panel as an individual-level surrogate for survival in metastatic castration-resistant prostate cancer. J Clin Oncol. (2015) 33:1348–55. 10.1200/JCO.2014.55.3487 25800753 PMC4397279

[27] Cortese R Kwan A Lalonde E Bryzgunova O Bondar A Wu Y. Epigenetic markers of prostate cancer in plasma circulating DNA. Hum Mol Genet. (2012) 21:3619–31. 10.1093/hmg/dds192 22619380

[28] Liu M Wang X Wang L Ma X Gong Z Zhang S. Targeting the IDO1 pathway in cancer: from bench to bedside. J Hematol Oncol. (2018) 11:100. 10.1186/s13045-018-0644-y 30068361 PMC6090955

[29] Stone TW Darlington LG. Endogenous kynurenines as targets for drug discovery and development. Nat Rev Drug Discov. (2002) 1:609–20. 10.1038/nrd870 12402501

[30] Uyttenhove C Pilotte L Théate I Stroobant V Colau D Parmentier N. Evidence for a tumoral immune resistance mechanism based on tryptophan degradation by indoleamine 2,3-dioxygenase. Nat Med. (2003) 9:1269–74. 10.1038/nm934 14502282

[31] Mengus C Le Magnen C Trella E Yousef K Bubendorf L Provenzano M. Elevated levels of circulating IL-7 and IL-15 in patients with early stage prostate cancer. J Transl Med. (2011) 9:162. 10.1186/1479-5876-9-162 21943235 PMC3191336

[32] Chow MT Moller A Smyth MJ. Inflammation and immune surveillance in cancer. Semin Cancer Biol. (2011) 22:23–32. 10.1016/j.semcancer.2011.12.004 22210181

